# Use of monosodium-glutamate as a novel dietary supplement strategy for ovarian stimulation in goats

**DOI:** 10.1590/1984-3143-AR2023-0094

**Published:** 2023-11-10

**Authors:** Anne Caroline Santos Soares, Juliana Paula Martins Alves, César Carneiro Linhares Fernandes, Maria Raquel Lopes Silva, Alfredo José Herrera Conde, Dárcio Ítalo Alves Teixeira, Davide Rondina

**Affiliations:** 1 Faculdade de Veterinária, Universidade Estadual do Ceará, Fortaleza, CE, Brasil; 2 Faculdade de Veterinária, Universidade de Fortaleza, Fortaleza, CE, Brasil

**Keywords:** goat, glutamate monosodium, follicle, ovary, Doppler, diet supplementation

## Abstract

This study aimed to investigate the reproductive effects of adding monosodium glutamate (MSG) to the diet of goats. Eleven adult goats received synchronized estrus and follicular waves using three prostaglandin analog injections every seven days. Goats allocated to individual pens received 1 g/kg BW of MSG in their diet for 23 days (MOGLU group, n = 6), whereas the control group (n = 5) maintained the base diet. The supplemented animals showed an increase in dry matter intake (P < 0.0001) and a reduction in heart rate (P < 0.05), respiratory rate, and ruminal movement (P < 0.001). Surface and rectal temperatures were higher in the MOGLU group, (P < 0.0001) with a significant increase in the afternoon. There was an increase (P < 0.05) in the frequency of behaviors related to rumination, defecation, and urination in the MOGLU group, and a reduction in behaviors associated with stress (P < 0.05). No differences were observed in the plasma levels of proteins, albumin, urea, cholesterol, or triglycerides. Glucose levels were lower (P < 0.05) in the MOGLU group, which also showed increased glutathione peroxide levels during the induction of ovulation. Supplemented animals recorded a larger number (P < 0.05) of follicles throughout the experimental period and higher intraovarian blood perfusion (P < 0.05) during ovulation induction. We conclude that MSG exerts a positive effect on the reproductive response in goats and therefore represents an effective nutritional supplement.

## Introduction

AA plays an important and recognized role in several reproductive functions in ruminants. For example, supplementation with rumen-protected methionine helps maintain pregnancy in multiparous cows and improves embryonic survival (Ayyat et al., 2021). The use of citrulline in the diet of ewes favors increased plasma concentrations of gonadotropin-releasing hormone (GnRH), luteinizing hormone (LH), and follicle-stimulating hormone (FSH) (Yan et al., 2023). In addition, the use of AA can influence the hypothalamic-pituitary-gonadal system through excitatory neurotransmitters, such as glutamate, which directly act on the neurons involved in the synthesis of reproductive hormones, influencing puberty (Meza-Herrera et al., 2014a).

The role and mechanism of glutamate in reproduction is well defined. Several studies have shown that the use of intravenous glutamate influences the return to reproductive cyclicity of goats in seasonal anestrus, with an increase in LH secretion peaks, ovarian activity, ovulation rate, number of antral follicles, and the onset of early puberty (Meza-Herrera et al., 2020; Luna-García et al., 2022).

Despite this evidence, the intravenous administration of glutamate for reproduction has limitations from both practical and practical perspectives. However, the main limitation of the inclusion of any amino acid supplement in ruminant diets is the ruminal environment and the extensive degradation and remodeling of dietary proteins (Gilbreath et al., 2021). Once it arrives in the rumen, the protein provided in the diet is hydrolyzed by proteases and peptidases into peptides and AA. The AAs are used and degraded by microorganisms, producing microbial proteins. This process provides more than 50% of absorbed AA in ruminants but makes it difficult to identify the AA actually available for subsequent absorption in the duodenum (Rossi et al., 2007).

However, Gilbreath et al. (2021) reviewing their recent results (Gilbreath et al., 2019; Gilbreath et al., 2020) described how some AAs, such as L-citrulline and L-glutamate, have a superior escape capacity than ruminal microorganisms, and therefore, they are not necessarily protected in the ruminal environment. These results are promising for the potential use of AA as a direct supplement in feed, a fact that would allow an increase in its use and practicality in ruminant diets. Thus, both L-citrulline and L-glutamate are potential candidates for use in diets in a functional way (Gilbreath et al., 2021). L-citrulline positively affects milk production (Wu et al., 2022). L-glutamate is required for the synthesis of antioxidant components such as glutathione (Newsholme et al., 2003) and improves digestive functions (Li et al., 2022).

Recently, Hisadomi et al. (2022) used rumen-protected monosodium glutamate (MSG) in dairy cows diet during the periparturient period, which increased digestive capacity and feed intake and decreased body fat and protein mobilization immediately after calving. Padunglerk et al. (2017) replaced soybean meal with MSG in the diet of dairy cows and found no negative effects on dietary dry matter intake, digestibility, and milk production and composition. In goats, Rukboon et al. (2018) associated MSG with cassava pulp and reported an increase in dietary dry matter intake and animal performance. These results pave the way for the possibility of including glutamate in the diet of ruminants in the form of salts, such as MSG, a well-known flavor enhancer that is widely used in human food (Thuy et al., 2020). Furthermore, the effects of MSG on reproductive activity should be evaluated, considering the lack of data in this regard. However, until now, limited studies in ruminants have focused on productive aspects, and not is known about the impact of this product on the reproductive response.

We hypothesized that the ruminal escape of glutamate is effective and allows direct stimulation of the ovarian response in goats and that it can be included in the diet in the form of MSG. Thus, the present study aimed to investigate the effects of including MSG in the diet of goats on ovarian follicular dynamics, ovulation rates, oxidative stress, and intraovarian blood perfusion. This study also verified the acceptance of the product in animals through clinical-behavioral observations, physiological-environmental parameters, and plasma metabolic profiles.

## Materials and methods

### Location, animal, and experimental treatments

This study was conducted on a farm of the School of Veterinary Medicine, Ceará State University, located in the equatorial zone (4°2ʹ23ʺ S and 38°38ʹ14ʺ W) of Brazil. This area is characterized by a constant photoperiod regimen and has a warm, tropical, and subhumid climate.

Eleven adult, pluriparous, non-pregnant, crossbred Anglo-Nubian goats of similar (P > 0.05) age (4.09 ± 0.8 years; overall mean ± SD), body weight (37.00 ± 5.00 kg), and body condition (2.8 ± 0.1; from 1 to 5 score) were used. The animals received a diet composed of a total mixed ration (TMR) prepared in water solution and based on chopped elephant grass plus concentrated (ground corn grain, 24%; wheat white, 12.8%; soybean meal, 1.2%; and mineral and vitamin mixture, 2.0%, based on dry matter of diet), provided in amounts to satisfy the nutritional requirements of adult non-dairy maintenance goats (NRC, 2007). The experimental animals were kept in individual shelter-clayed open boxes with free access to mineral supplements and water. The TMR was administered twice daily. The refused feed was collected daily and weighed weekly to determine individual intake and diet adjustment. Before the experiments, the goats underwent a 30-day management adaptation after receiving endo-and ectoparasitic treatments and were vaccinated against clostridiosis. Throughout the pre-experiment, cyclicity and ovarian function were monitored by ultrasound examinations and sexual receptivity in mature fertile males according to Fernandes et al. (2018).

**Figure 1:**
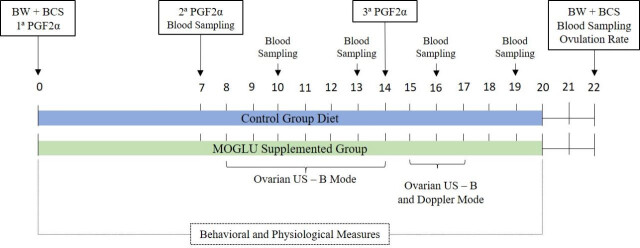
General experimental design including dietary treatments, hormonal protocol, ultrasonography analysis, behavioral and physiological measure.

The goats had synchronized estrus and follicular waves as described by Viñoles et al. (2010) through three injections of 100μg of the prostaglandin analog (PGF2 alpha) cloprostenol sodium (SINCROCIO®; Ourofino; Brazil) at intervals of seven days (Figure 1). The third injection was given to promote estrus and ovulation simultaneously in all goats. During hormonal treatment from day 0 to 20, 1 g/kg BW of MSG (DellaTerra®, São Paulo, Brazil; 99% purity) was added daily to the diet concentrate of six animals (MOGLU Group, n = 6). In the control group (n = 5), the initial TMR was maintained (Figure 1).

### *In vivo* performance and carcass markers

On days 0 and 22 of the study, goats were weighed, and fat and muscle mass were measured (Figure 1) using a B-mode ultrasound device with a 5 MHz linear probe (model Z5 Vet; Mindray Bio-Medical Electronics Co., Shenzhen, China). The depth of the Longissimus dorsi muscle and the thickness of the subcutaneous fat of the loin were measured between the third and fourth lumbar vertebrae (Teixeira et al., 2008), as well as the thickness of the subcutaneous fat deposits at the third sternebrae (Alves et al., 2019). To estimate visceral fat, the thickness of renal fat behind the 13th rib was measured (Härter et al., 2014). A convex transducer with a frequency of 3.5 MHz was used for the renal imaging. Images were captured in triplicate and measured using previously calibrated ImageJ software (ImageJ, National Institutes of Health, Millersville, USA).

### Animal response measurements

#### Thermo-physiological measurement

Physiological and environmental parameters were measured twice daily at 7:00 am and 2:00 pm during the entire glutamate administration period (Figure 1). Rectal temperature (RT) was measured using a digital clinical thermometer (G Tech®, Hangzhou Sejoy Electronics, Hangzhou, China). Skin surface temperature (ST) was calculated as the mean of three different shorn site measurements: head, loin, and rump, using an infrared thermometer (AK32®, AKSO, São Leopoldo, Brazil). The heart rate (HR) was assessed with the animal in a standing position using a stethoscope (3M Littmann®, Master Classic II™, St. Paul, USA) positioned on the left side of the thorax, close to the heart. The pulse sound was recorded for 15 s, and the resulting value was multiplied by four to obtain the frequency in beats per minute. In sequence, the respiratory rate (FR) was evaluated with a stethoscope for the auscultation of the pulmonary sounds that were counted for 15 seconds, and at the end they were multiplied by four, resulting in the frequency of breaths per minute. Subsequently, ruminal movements were evaluated by positioning the stethoscope in the left paralumbar fossa and counting ruminal movements during 2 min of auscultation. Ambient temperature and relative humidity were recorded using a portable thermohygrometer (AK624®, AKSO, São Leopoldo, Brazil).

#### Behavioral evaluation

During the period of glutamate administration, the animals were examined regarding their behavior (Figure 1) using the methodology adapted from Leme et al. (2013), which consisted of a 30-minute sampling interval directly in two moments of the day, at 7:00 am and 2:00 pm, totaling 1 hour of daily evaluation. According to Bateson and Martin (2007), dose behaviors were recorded as activities (water consumption, urination, defecation, rumination, diet consumption, and idleness) and behaviors of punctual events related to stress indicators (vocalization, isolation, headbutting, sniffing, biting, and moving the head, ears, or thoracic limbs). Trained observers performed all the recorded activities.

### Ultrasonography analysis

#### Ovarian follicular dynamics and ovulation rate

Ultrasonography was performed once a day from the 8th to the 14th day and twice a day from the 15th to the 17th day (Figure 1). Ovarian images were captured using a B-mode ultrasound device and a 5 MHz linear transrectal probe (model Z5 Vet; Mindray Bio-Medical Electronics Co., Shenzhen, China). A video was recorded per ovary for each animal, and images were captured and analyzed using previously calibrated Image J software. An ovarian follicular wave is defined as the appearance of a cluster of small follicles (3 mm to < 4 mm) that develop into one or more large follicles (≥ 5 mm) (Ginther and Kot, 1994). An intermediate diameter (≥ 4 mm to < 5 mm) was defined as a medium-sized follicle. The ovulation rate was determined by observing the collapse of large follicles (≥ 5 mm), followed by the evaluation of the presence and count of corpora lutea in the same location eight days later, following the methodology of Viñoles et al. (2010), and by laparoscopy on the 22nd day, through a corpus luteum classification (Guido et al., 2003).

#### Intraovarian blood flow

The evaluations of intraovarian blood flow were performed from the 15th to 17th days of the experiment (Figure n 1) using a color Doppler ultrasound device equipped with a 7.5 MHz linear transrectal probe (model Z5 Vet; Mindray Bio-Medical Electronics Co., Shenzhen, China). Equipment settings were kept constant throughout the study, with a Doppler sampling frequency (PRF) of 1.0 kHz, depth of 6.5 cm, and color gain of 100%. Using ImageJ software, a quantitative analysis of the colored pixels present in the Doppler area was performed to evaluate the intraovarian blood flow in the images, following the protocol described by Cavalcanti et al. (2022).

### Blood sampling, metabolite, and glutathione peroxidase assays

Every third day, between days 7 and 22 of the study, blood samples were collected in the morning before food administration using heparinized vacutainer tubes (Firstlab®, Disera Tıbbi Malzeme Lojistik San. Tic. A.Ş, Izimir, Turkey). The samples were centrifuged at 3000 rpm for 10 minutes to separate the plasma, which was then stored at -20°C. Using an automated biochemical analyzer (Mindray BS 120, Mindray Biomedical Electronics Co., Shenzhen, China), plasma concentrations of glucose, cholesterol, triglycerides, total proteins, albumin, urea, and glutathione peroxidase (GPx) were determined using commercial kits (Bioclin , Quibasa, Minas Gerais, Brazil) with sensitivities of 1.31 mg/dL, 1.472 mg/dL, 2.845 mg/dL, 0.043 g/dL, 0.0327 g/dL, and 1.514 mg/dL, respectively. GPx was analyzed using a commercial kit (Ransel, Randox Laboratories®, Crumlin, UK) with a sensitivity of 75 U/L.

### Statistical analysis

Statistical analyses were performed using Statistica Software version 13.4.0.14 (2018; TIBCO Software, Inc., Palo Alto, CA, USA). The data were initially verified for mathematical assumptions using the Kolmogorov–Smirnov and Bartlett’s tests. If these conditions were not met, a log10x transformation was applied.

All descriptive data were analyzed using the MIXED procedure for repeated-measures analysis of variance (ANOVA) with animals as a random effect. The factors used in the model for feed intake and physiological and behavioral responses included group (Control, MOGLU), time of supplementation (1,2 and 3 weeks), daily reading (morning reading, MR and afternoon reading, AR), and interactions. For the other parameters, the effects were group, the interval of the sample used (time), and interactions. All pairwise comparisons were performed using the Newman–Keuls post-hoc test. The ovulatory rate effect of the groups was analyzed using the Kruskal-Wallis ANOVA test.

### Ethical statement

All procedures followed in this study were approved by the Ethics Committee on Animal Experimentation of the UECE (No. 490620/2020, CEUA-UECE).

## Results

### Body weight and carcass markers

Regarding live weight (Table 1), no significant effects were observed in relation to groups (P = 0.8365) or between weighing intervals (Effect time P = 0.8868). There were also no differences between the groups and measurement intervals for ultrasonographic carcass markers, except for lumbar subcutaneous adipose deposits (Table 1), which were lower in animals supplemented with MSG (P = 0.0002).

**Table 1:** Body weight and carcass markers, in goats fed with a baseline diet or diet supplemented with monosodium glutamate.

**Parameters**	**Group**			**p Value**		
	**Control**	**MOGLU**	**SEM**	**Group**	**Time**	**G x T**
Body weight, kg	36.84	37.30	1.0151	0.8365	0.8868	0.7869
*Carcass Markers*						
SSFT, mm	12.46	11.40	0.3868	0.1722	0.8621	0.1212
SLFT, mm	4.02	3.62	0.0545	0.0002	0.8157	0.3360
KFT, mm	2.09	2.02	0.0421	0.3705	0.4812	0.3449
DL, mm	17.07	15.86	0.3519	0.0868	0.4124	0.3087

Abbreviations: BW, Body weight; SSFT, subcutaneous sternal fat thickness; SLFT, subcutaneous loin fat thickness; KFT, kidney fat thickness; DL, depth loin; Time, ANOVA effect for interval of sampled used.

### Alimentary, physiological, and behavioral responses

The environmental temperatures recorded during the experimental period were 28.44 ± 0.21 ºC and 28.51 ± 0.21 ºC for the minimum and maximum values, respectively. The minimum relative humidity recorded was 73.41 ± 0.92% and the maximum was 73.87 ± 0.91%. Significant variations (P < 0.0001) in environmental parameters were recorded between daily measurements, with an increase in temperature (+ 2.2 ºC) and a decrease in relative humidity (- 6.8%) in the afternoon reading. No changes were observed in these parameters during the supplementation period.

Table 2 illustrates the data relating to the animal response during the administration of MSG. Regarding dry matter intake and the amount of food refused, the MOGLU group showed higher values (P < 0.0001) when compared to the control group. There was a significant reduction for both groups in afternoon food intake (Effect daily reading P < 0.05), without interaction with the group. On the contrary, there was an interaction between the group and the weeks of glutamate administration (P < 0.0001) due to the dry matter reduction intake (- 16%) that occurred in the MOGLU from the first to the second week of supplementation. In this group, the consumption of food in the third week returned to values similar to the beginning of the experiment and significantly higher than the control group (P < 0.001) only in the measurement performed in the morning.

**Table 2:** Feed intakes, physiological and behavioral responses, in goats fed with a baseline diet or diet supplemented with monosodium glutamate.

**Parameters**	**Group**			**p Value**				
	**Control**	**MOGLU**	**SEM**	**Group**	**TS**	**DR**	**G x DR**	**G x TS**
***Feed Intake***								
DMI, g/kg MW	78.68	90.57	1.0224	< 0.0001	< 0.0001	0.0136	0.1337	< 0.0001
DMI, % BW	3.20	3.64	0.0435	< 0.0001	< 0.0001	0.0206	0.1764	< 0.0001
Refused feed, %	24.47	30.68	0.8544	< 0.0001	< 0.0001	0.0018	0.1854	< 0.0001
***Physiological response***								
HR, beat\min	66.24	64.79	0.4225	0.0364	0.0002	< 0.0001	0.2584	0.8049
RF, breaths\min	39.69	35.17	0.7582	0.0017	< 0.0001	< 0.0001	0.7205	0.9252
ST, ºC	31.31	32.12	0.0971	< 0.0001	0.0042	< 0.0001	0.0009	0.3260
RT, ºC	38.49	38.30	0.5349	< 0.0001	0.0438	< 0.0001	0.0001	0.2869
RM, (mov\min)	2.18	1.95	0.7269	0.0004	< 0.0001	0.0004	0.9909	0.7099
***Behavior response***								
Rumination, %	39.93	60.07	0.0466	0.0192	0.4517	0.0260	0.1864	0.0982
Idle, %	56.03	43.97	0.0464	0.1149	0.1045	0.1452	0.2866	0.2251
Drinking, %	50.00	50.00	0.0570	1.0000	0.1574	0.0078	0.1291	0.0510
Feeding, %	48.17	51.83	0.1164	0.4815	0.6197	0.0032	0.1866	0.3028
Urination, %	12.36	87.64	0.1117	0.0015	0.9040	0.9824	0.5454	0.9798
Defecation, %	28.46	71.54	0.0860	0.0152	0.2763	0.2153	0.2465	0.4613
Stress indicators, %	73.00	27.00	0.1329	0.0253	0.0293	0.3101	0.0401	0.1188

Abbreviations: DMI, dry matter intake; MW, metabolic weight; HR, heart rate; RF, respiratory frequency; ST, superficial temperature; RT, rectal temperature; RM, ruminal movements; TS, ANOVA effect for supplementation length (1,2 and 3 week); DR, ANOVA effect for daily reading measures (MR, AR).

All physiological parameters showed significantly lower mean values in the MOGLU group than in the control group (Table 2). Both groups exhibited an increase in physiological measurements in the afternoon. However, animals in the MOGLU group showed a greater increase (+ 2.6 ºC ST, + 0.8 ºC RT) in surface and rectal temperatures than those in the control group (Interaction G x DR, P < 0.001).

Regarding behavioral parameters (Table 2), the supplemented animals reported more intense activity concerning rumination, defecation, and urination (P < 0.05) than the control group. In contrast, stress indicators showed a reduction in the frequency of manifestations in the MOGLU group (P = 0.0253). For this parameter, there was an interaction between the group effects and time (Interaction G x DR P = 0.0401), due to the increase in indicators during the afternoon measurement in the control group (27.40% MR vs. 72.60% AR; P = 0.0425), which did not occur in animals from the MOGLU group (53.57% MR vs. 46.43% AR; P > 0.05). Therefore, in the afternoon, the nutritional groups differed significantly (P = 0.0340).

### Peripheral metabolites and GPx levels

Except for glucose, the plasma metabolite levels were similar between the groups (Table3). MOGLU treatment showed a lower mean glucose level and a significant interaction with time (P = 0.0040) as a function of reduced plasma concentrations of this metabolite in the MOGLU group between days 10 and 13 of supplementation. From the 14th day, plasma glucose levels returned to values similar (P > 0.05) to those in the control group. The peripheral concentration of GPx was higher in the MOGLU group (P = 0.0241). GPx levels increased in the supplemented animals from the 16th day of glutamate administration until the 22nd day, in contrast to what was observed in the control group (Interaction G x T P = 0.0230).

**Table 3:** Metabolites levels and glutathione peroxidase, in goats fed with a baseline diet or diet supplemented with monosodium glutamate.

**Parameters**	**Group**			**p Value**		
	**Control**	**MOGLU**	**SEM**	**Group**	**Time**	**G x T**
*Metabolites levels*						
Total protein, mg/dL	6.17	6.09	0.0888	0.6528	0.2862	0.0531
Albumin, mg/dL	2.42	2.30	0.0533	0.4790	0.0909	0.8301
Urea, mg/dL	22.00	22.22	0.5242	0.6228	0.0040	0.1820
Glucose, mg/dL	48.67	45.79	0.6724	0.0149	0.0575	0.0025
Cholesterol, mg/dL	64.93	58.00	1.6610	0.1093	0.8669	0.6219
Triglyceride, mg/dL	22.07	22.89	0.7265	0.6988	0.0129	0.0565
Glutathione peroxidase, U/L	330.23	339.69	2.1901	0.0241	0.5252	0.0230

Time, ANOVA effect for interval of sampled used.

### Reproductive response

Animals in the MOGLU treatment group had a greater (P < 0.01) number of small (< 3 mm) and total follicles before and after ovulation induction (Table 4). The number of large follicles (≥ 3 mm) was lower than that in the control group before ovulation (P = 0.0177), whereas no significant differences were observed between the two nutritional groups after ovulation induction. Differences were also observed between the two follicular classes (large vs. small), with a greater number (P < 0.05) of large follicles in the control group between the 7th and 14th day of supplementation, and after induction in MOGLU, the number of small follicles was higher than that in large follicles (P < 0.05). When comparing periods (before vs. after ovulation induction), there was a decrease in the number of large follicles in the control group (2.16 vs. 1.70; P < 0.05) and an increase in the total number of follicles(3.69 vs. 4.25; P < 0.05). The maximum follicular diameter in the MOGLU group was smaller (P = 0.0177) than the 7th and 14th days compared to the control group. However, the supplemented group exhibited an increase in follicle size between the periods analyzed (4.54 mm vs. 4.91 mm; P < 0.05).

**Table 4:** Reproductive response in goats fed with a baseline diet or diet supplemented with monosodium glutamate.

**Parameters**	**Group**			**p Value**		
	**Control**	**MOGLU**	**SEM**	**Group**	**Time**	**G x T**
Follicles traits before ovulation induction*						
Follicles < 3 mm, n\ovary	0.87a	1.95	0.1303	< 0.0001	0.6887	0.9983
Follicles ≥ 3 mm, n\ovary	2.16bA	1.74	0.0776	0.0068	0.2738	0.6160
Total follicles, n\ovary	3.03	3.69A	0.1376	0.0177	0.2881	0.9961
Max follicle size, mm	4.85	4.54A	0.0717	0.0274	0.8795	0.4340
*Follicles traits after ovulation induction* **						
Follicles < 3 mm, n\ovary	1.27	2.33a	0.1028	0.0104	0.9462	0.7077
Follicles ≥ 3 mm, n\ovary	1.70B	1.92b	0.1167	0.2913	0.8306	0.1748
Total follicles, n\ovary	2.97	4.25B	0.1290	0.0010	0.8967	0.9887
Max follicles size, mm	4.64	4.91B	0.1885	0.2611	0.8912	0.8069
Intraovarian blood flow area, %\goat	1.23	2.35	0.2554	0.0227	0.7573	0.9143
Ovulatory rate, n	1.00	0.67	0.2263	0.4926	-	-

* Follicles ultrasonography traits measured on the 7^th^ Day to the 14^th^ Day of MOGLU supplementation; **Follicles traits and Intraovarian Doppler area, measured by ultrasonography in the 48 hours after ovulation induction; Time, ANOVA effect for interval of assessment used. a,b P < 0.05 differences between follicular classes in the same period (before or after ovulation induction). A, B differences between periods (before and after ovulation induction).

MOGLU treatment also resulted in a greater (P = 0.0227) intraovarian Doppler area than that in the control group (Table 4). Ovulation rates were similar between the groups (P = 0.4926).

## Discussion

### Animal acceptance

During the experiment, the administration of MSG did not result in specific clinical signs in the supplemented animals, which would indicate an anomalous response to the product, even though the dose was within the maximum limit for the appearance of possible physiological alterations (Walker and Lupien, 2000), according to the dosage used for dairy cattle by Padunglerk et al. (2017). In contrast, a reduction in behavioral indicators of stress was observed. The inclusion of MSG substantially stimulated consumption and better use of the diet, which was reflected in an increase in some behavioral traits in this group, such as the frequency of rumination, defecation, and urination. The greater palatability of the feed is an expected result owing to the characteristics of MSG, which opens up the possibility for future studies in goats with testing levels and application times that are different from those used in the present study.

MSG is the sodium salt of glutamic acid, which has a crystallized form and is commonly used in the food industry as a flavor enhancer. It is more consumed in the Asian continent, contributing to the improvement of flavor characteristics, such as complexity and smoothness; thus, it is considered the fifth flavor called umami (Thuy et al., 2020). In animals, its use in some species, such as mice, has caused focal necrosis in the arcuate nucleus of the hypothalamus, with young animals being more susceptible (Walker and Lupien, 2000). Rezaei et al. (2022) demonstrated that MSG moderately decreased food consumption in pigs, which was related to increased consumption of sodium. However, in goats, the inclusion of cassava pulp mixed with MSG byproducts increased feed intake (Rukboon et al., 2018).

In this study, despite higher food intake, no variations in weight or main carcass markers were observed during the experimental interval, nor were there any critical changes in the peripheral concentrations of the protein and energy metabolic profile. These metabolite levels are in agreement with those reported by Dutra et al. (2022) and Hernandez et al. (2020) in goats. Our protein profile data did not suggest an escape of glutamate from ruminal microbial action, as reported by some authors (Gilbreath et al., 2019). However, it is worth mentioning that this result was unexpected because the present study was not designed to obtain this information. Previous research has shown that the intravenous administration of glutamate in goats does not modify the profile of total proteins and urea (Meza-Herrera et al., 2014b). According to reviews by Gilbreath et al. (2021), the administration of amino acids in isolation is not capable of altering the protein profile, as their metabolism occurs more slowly than supplying a set of AA.

Regarding physiological parameters, the groups recorded values within the range expected for the species in this region (Rondina et al., 2004; Ribeiro et al., 2018), although the supplemented group reported a reduction in heart and respiratory rates and ruminal movements. As expected, the physiological response of the animal was highly influenced by the increase in environmental temperature during the afternoon measurements which represented the hottest moment of the day (Rondina et al., 2004). However, the data obtained focused on the greater sensitivity of animals in the MSG group which recorded a change in food consumption that occurred at the same time in the second and third weeks of supplementation and was associated with significant changes in surface and rectal temperatures during the hottest period of the day. However, an increase in temperature is related to an increase in endogenous heat production, which in turn is subordinated to dietary intake and represents an animal response to the new energy balance imposed. In our study, the addition of MSG, despite transient changes, proved effective concerning diet consumption.

### Reproductive response

The results of the study confirmed the initial hypothesis that the addition of MSG to the diet induced a reproductive response in goats, stimulating an increase in the follicular population at the ovarian level and blood perfusion at the intraovarian level during ovulation. Specifically, the data showed that the use of 1 g/kg BW of this amino acid supplement promoted a substantial increase in small follicles (< 3 mm) during the second week of supplementation, and this phenomenon continued after ovulation induction. Despite not having verified the advantages in the ovulation rate between the groups, there was also a positive evolution in the follicular dynamics between the second week of supplementation and the period of ovulation induction. During this transition, there was a progression in follicular size (≥ 3 mm) in contrast to what occurred in the control treatment. Furthermore, these processes were accompanied by the acquisition of a more intense blood supply, as evidenced by the intraovarian Doppler results in the supplemented animals, as well as by an increase in peripheral oxidative stress markers.

In in vitro studies, it was observed that follicular cultivation with the addition of amino acids such as arginine, lysine, and methionine enabled the activation of primordial ovarian follicles in bovines, as observed by Sakaguchi et al. (2023). This effect may be related to the inhibition of the homologous tensin and phosphatase pathways (PTEN), which are the main negative regulators of phosphoinositide 3-kinase (PI3K), thus contributing to the perpetuation of dormancy in primordial follicles. Furthermore, Alborzi et al. (2020) found that, when assessing the activation of primordial follicles, the positive effect of glutamine, the primary precursor for glutamate synthesis, was partially attributed to the upregulation of Pi3k which activates the mammalian target pathway of rapamycin (mTOR ), stimulating the activation of these follicles.

The process of follicular activation and growth requires a greater blood supply, and consequently, an increase in intraovarian vascularization to promote development and maturation, as this supply is reflected in the greater circulation of nutrients, oxygen, and gonadotropic and steroidal hormones (Acosta et al., 2003). However, during this phase, due to the higher proliferation rates of theca and granulosa cells, there is an increase in intrafollicular oxidative components, which increases reactive oxygen species (ROS) that, at excessive levels, cause intracellular damage, affecting follicular and oocyte quality (Yuan et al., 2022).

However, cells have a defense system composed of enzymes such as glutathione peroxidase (GPx), which neutralizes peroxide free radicals, contributing to the maintenance of redox balance and reduction of oxidative stress (Pei et al., 2023). During MSG supplementation in goats, there was an increase in GPx activity, a phenomenon attributed to increased protection against ROS produced during oxidative stress, although some studies found that the use of the same supplement gave an opposing result (Ahmed et al., 2019). In addition, the chronic use of free glutamate, administered as tablets in rats, increased the antioxidant enzymes GPx, SOD, and CAT, in addition to an increase in glutathione (GHS) and total proteins with a decrease in lipid peroxidation (Tabassum et al., 2020). Furthermore, studies on glutamine supplementation in small ruminants have shown increased GPx activity, favoring an increase in antioxidant capacity and reducing oxidative stress (Feyz et al., 2021). Supported by the fact that glutamate is a product of glutamine metabolism and participates in the biosynthesis of glutathione, its importance, along with cysteine and glycine (Wang et al., 2021), in antioxidant mechanisms is justified.

In goats, one of the known effects of glutamate is the gonadotrophic stimulus, since it induces a significant increase in the number of antral follicles when administered intravenously during the follicular phase (Meza-Herrera et al., 2014b) due to increased glutamatergic transmission via Kiss 1 neurons directly to GnRH neurons. This mechanism explains the high number of < 3 mm follicles observed in this experiment, which can be attributed to the intense follicular recruitment process. With regard to the regulation of systems involving gonadotropic hormones, amino acids such as glutamate act as excitatory neurotransmitters of Kiss1 neurons and actively participate in the pulsatility mechanisms of GnRH (Lass et al., 2022). This results in the release of luteinizing hormone (LH) and follicle-stimulating hormone (FSH) gonadotropins as these neurons express glutamate receptors (Iremonger et al., 2010). During the estrous cycle in goats, continuous selection and development of ovarian follicles occur as a result of the proliferation and differentiation of follicles stimulated by insulin-like factor-1 (IGF-1) (Ipsa et al., 2019). In goats, studies have shown that IGF-1 is related to the activation of primordial follicles as well as the development of primary and secondary follicles (Magalhães-Padilha et al., 2012). Because the IGF-1 protein is expressed at all stages of follicular development and is still associated with FSH, it promotes granulosa cell proliferation, cell differentiation, and antrum formation (Monte et al., 2019).

## Conclusions

We conclude that MSG, under the conditions used in the present study, represents an effective option for nutritional supplementation in goats. Glu stimulates animal feed intake, induces ovarian follicular population growth, and improves intraovarian blood flow perfusion during ovulation. Despite these results, the higher sensitivity of supplemented animals to the increase in environmental temperature in our study requires more data to ascertain the feasibility of using MSG in areas with a high-temperature climate using different times and dosages than those proposed here. Therefore, the authors recommend further studies to be undertaken to understand the effect of this supplement on animal responses.

## Data Availability

All data generated and analysed during this study are included in this published article.
